# Clinical Pathways in Managing High-Caries Children with Adverse Dental Histories

**DOI:** 10.3390/medicina61122105

**Published:** 2025-11-26

**Authors:** Flora Hashemi, Ahmad Al Masri, Christian H. Splieth, Julian Schmoeckel

**Affiliations:** 1 Department of Pediatric Dentistry, University Medicine Greifswald, 17475 Greifswald, Germany; flora.hashemi@uni-greifswald.de (F.H.); ahmad.almasri@uni-greifswald.de (A.A.M.); splieth@uni-greifswald.de (C.H.S.); 2 Department of Orthodontics, University Medicine Greifswald, 17475 Greifswald, Germany

**Keywords:** dental caries, dental anxiety, conscious sedation, anesthesia, general, behavior control, child

## Abstract

*Background and Objectives*: Dental treatments of children with previous negative dental experiences are challenging and frequently require sedation or general anesthesia. Therefore, this study aimed to identify treatment pathways and factors associated with dental treatment under general anesthesia, nitrous oxide sedation, and chairside management among pediatric dental patients with adverse dental experiences. *Materials and Methods*: A retrospective cohort analysis was conducted on all new children aged 2–12 years with negative dental experience and treatment need visiting the Pediatric Department of the Dental Clinic, University of Greifswald, between 1 January and 31 December 2021. *Results*: Of the 640 newly presenting patients, 78 cases met inclusion criteria and were analyzed (mean age 6.7 ± 2.19 years, baseline dmft 4.5 ± 3.0 and DMFT 0.6 ± 1.2), with 88.5% having caries as chief complaint and 61.5% having a referral from other dentists. Eventually, only 25.6% received general anesthesia (mean age 5.1 ± 1.9 years, mean dt 5.6 ± 2.6), while 56.4% were managed with nitrous oxide sedation (mean age 7.3 ± 1.7 years, mean dt 3.3 ± 2.4), and 18% with chairside treatment (mean age 6.9 ± 2.8 years, mean dt 1.9 ± 2.4). Children of younger age, with higher dmft/dt scores and lower behavior ratings, were more frequently treated under general anesthesia. However, none of these variables could be identified as independent predictors for treatment under general anesthesia. *Conclusions*: Minimally invasive caries management, combined with appropriate behavior guidance and conscious sedation when indicated, can drastically reduce the need for general anesthesia in children, even among those with dental anxiety or previous negative dental experience.

## 1. Introduction

Despite widespread preventive measures and universal health coverage in Germany, caries in primary teeth persists in a polarized manner. Among three-year-old and 6–7-year-old children with caries experience (SaC-Index), the mean dmft remains 3.6 and 3.96, respectively, with 73.9% and 42.5% of affected teeth remaining untreated [1]. Globally, the burden of early childhood caries (ECC) is substantial: a meta-analysis of 100 studies from 49 countries reported a pooled ECC prevalence of approximately 49%, with 42.7% of caries in children aged 1–9 years remaining untreated [2]. Untreated dental caries negatively impacts children’s general health and quality of life [3,4,5,6,7,8,9], emphasizing the need for effective management in primary dentition.

Previous negative dental experiences are among the most significant contributors to dental fear and anxiety (DFA) [10,11,12,13], which in turn can lead to poor cooperation and avoidance of treatment [13,14]. This is particularly concerning in very young children with extensive ECC and prior negative experiences [15], who often require complex and extensive dental interventions frequently with advanced behavior management methods [16,17].

We hypothesize that younger age, low cooperation scale, higher numbers of decayed teeth (dt), and a dmft index greater than the child’s age are key predictors for requiring dental treatment under general anesthesia (GA). While GA allows completion of treatment in uncooperative children, long-term benefits are greater when negative experiences are addressed and overcome [18,19]. Specialized pediatric dentists can facilitate positive behavioral outcomes through tailored behavior management, minimally invasive interventions, and conscious sedation, fostering voluntary cooperation and reducing the impact of prior traumatic experiences [20,21].

This study, therefore, aims to analyze the treatment pathways of children presenting to a pediatric specialist dental center and to identify predictors of treatment under GA in this selected patient sample. The findings may guide general dentists in selecting appropriate treatment approaches and referral decisions, ultimately helping to prevent negative dental experiences in pediatric patients.

## 2. Materials and Methods

### 2.1. Study Design and Ethics

This study was designed as a descriptive–analytical retrospective cohort evaluation of a full year in the department of Pediatric Dentistry at UMG (Internal Regulation No. BB 028/16). On the first visit to the pediatric dental clinic, parents or legal guardians completed and signed a case history form that includes explicit consent for the use of anonymized data for research purposes. Only data of patients with signed case history forms were included in this study. All data were retrieved pseudonymized (using a unique patient ID) and anonymized prior to analysis to protect patient privacy.

During the preparation of this manuscript/study, the authors used ChatGPT 5 and DeepL in the manuscript to improve grammar, spelling, wording and language clarity. The authors have reviewed and edited the output and take full responsibility for the content of this publication.

### 2.2. Sample Size

As neither global nor national data are available regarding the prevalence of dental treatment under GA in children, particularly those with negative dental experiences, we estimated an overall GA prevalence of 5% among all new patients in our pediatric dental clinic. For the purpose of stratification, we assumed that approximately 10% of children within our study sample would require GA (representing a higher-risk subgroup), compared to an estimated 3% prevalence of GA among all new patients. Based on these assumptions, a sample size of 70 participants was necessary to achieve a 95% confidence level with a 5% margin of error (alpha) and 80% power.

### 2.3. Patient Selection

The records of all patients who visited the Department of Pediatric Dentistry at the University of Greifswald between 1 January 2021 and 31 December 2021 were screened for eligibility according to the following criteria:

#### 2.3.1. Inclusion Criteria

- First visit to the department of Pediatric Dentistry in 2021 (January–December);
- Child-specific case history fully completed and signed by parents/legal guardians;
- Age between 2 and 12 years (as dental GA is covered by German health insurance until the age of 12 years);
- Reported history of negative dental experience;
- Dental treatment needs (dt/DT > 0);
- Completion of dental treatment within a maximum of 18 months.

#### 2.3.2. Exclusion Criteria

- Incomplete or unsigned case histories;
- Absence of complete documentation in patient’s digital records;
- Absence of child-specific case histories;
- No further appointments after the first session;
- Emergency treatment needs only.

### 2.4. Initial Visit Protocol in the Department of Pediatric Dentistry of the Dental Clinic of the University Medicine Greifswald

Each new pediatric patient visiting the Department of Pediatric Dentistry for the first time receives a child-specific case history form at the reception. This form is completed and signed by the parents or guardians and provides essential information regarding the child’s demographic profile, behavioral characteristics, medical and dental history, and chief complaint prior to entering the treatment room.

Upon entering the treatment room, the dentist introduces him or herself and invites the children to introduce themselves and state the reason for the visit. This initial interaction allows the clinician to assess the child’s level of cooperation, psychological state, and cognitive abilities. Parents are subsequently asked about the chief complaint, their expectations, any referral information, and their general attitudes towards routine dental check-ups, prophylaxis, and oral hygiene practices.

The dental examination begins with basic behavior management techniques (BMT), including the tell–show–do approach, interactive communication, locus of control and distraction using visual aids (e.g., a television screen on the ceiling above the dental chair). Oral hygiene is assessed using plaque-disclosing agents. Based on the findings, the child and parents receive personalized oral hygiene instructions, including practical toothbrushing exercises, and a professional topical fluoride gel application is performed using a handpiece with rotating brush.

If necessary, and in most cases of the study sample radiographic examinations are performed. Based on the clinical and radiographic findings, treatment planning and consultations are conducted, including a discussion with the parents regarding the proposed treatment and the setting for future sessions leading to decision taking with informed consent.

This initial visit allows the dentist to evaluate the child’s oral hygiene status, parental attitudes, and the child’s level of cooperation and behavior, all of which contribute to the decision-making process regarding the appropriate treatment setting. For potentially cooperative but anxious children, one or more desensitization sessions are scheduled on a private-pay basis (low symbolic cost) prior to initiating definitive treatment. In cases requiring emergency care, the decision to proceed is based on the child’s level of cooperation during the visit [22].

### 2.5. Data Collection

Data were collected from patients’ case history forms, completed by parents at the first visit, and from the digital records of children treated at the Department of Pediatric Dentistry, University Medicine Greifswald.

The following information was extracted from the case history forms: registration date, birth date, medical and dental history, chief complaint, reason for referral (if applicable), history of negative dental experiences, and parents’ agreement to either leave the treatment room during procedures (if necessary) or to pay for an additional desensitization visit.

Additional data were retrieved from the digital dental records, including gender, type of visit (emergency or non-emergency), presence of dental pain at the first visit, patient cooperation (assessed using the Frankl scale), and dt/DT and dmft/DMFT scores at the initial visit. The DMFT/dmft indices were calculated according to WHO criteria [23] based on the recorded number of decayed (d/D), missing (m/M), and filled (f/F) teeth. Only cavitated teeth were marked as decayed. Waiting time was calculated as the duration between the registration date and the next dental visit at the clinic. The number of desensitization visits needed was recorded based on documentation from the digital records.

According to the patients’ documentation, children were categorized into three groups based on the treatment setting: (1) general anesthesia (GA), (2) conscious sedation with nitrous oxide (N_2_O), or (3) chairside treatment without sedation. As this study was a retrospective analysis based on information extracted from patient records, the researchers had no influence on the selection of the treatment modality. The treatment setting had been determined prior to the initiation of dental care by a pediatric dentist, according to the severity of the required dental procedures and the child’s level of cooperation following desensitization visits and the application of appropriate behavior management techniques.

The type and number of performed dental treatments were collected, including fillings, stainless steel crowns (SSC), extractions, pulp therapies, injections, silver diamine fluoride (SDF) applications, non-restorative cavity control (NRCC), and fixed or removable space maintainers.

The type and number of dental procedures performed from the first visit till maximum 18 months after the first treatment session, as well as updated dt/DT and dmft/DMFT scores after treatment completion, were recorded based on information documented in their digital dental records. Treatment was considered completed if the dt/DT score was 0, if the remaining cavitated carious lesions were arrested through NRCC and/or SDF, if the dental treatment requested in the referral had been performed and the patient was referred back to the referring dentist, or if the next scheduled appointment was a routine check-up.

### 2.6. Data Analysis

Data were entered and organized using Microsoft Excel. The final dataset was exported to SPSS software, version (30.0) (IBM Corp., Armonk, NY, USA) for statistical analysis. Continuous data including age, dmft, dt, DMFT, and DT were reported in means and standard deviations (SD), while categorical data included gender, presence of known general diseases, chief complaint, emergency at the first visit, reason for referral, cooperation level (Frankl scale), dental pain history or pain at the first visit, parents’ willingness to cooperate, and treatment type were reported in frequencies and percentages.

The Chi-Square test was used to compare categorical variables among the three groups of treated children. The Shapiro–Wilk test was performed to assess the normality of distribution of numerical data. For variables with a normal distribution, one-way ANOVA was conducted to assess differences among the groups. For non-normally distributed variables, the Kruskal–Wallis test was performed. Logistic regression analysis (univariate score tests and multivariate logistic regression model) was performed to identify whether any independent variables or a combination could predict the necessity of dental treatment under general anesthesia (GA).

A *p*-value of less than 0.05 was considered as threshold for statistically significant finding.

## 3. Results

**Figure 1:**
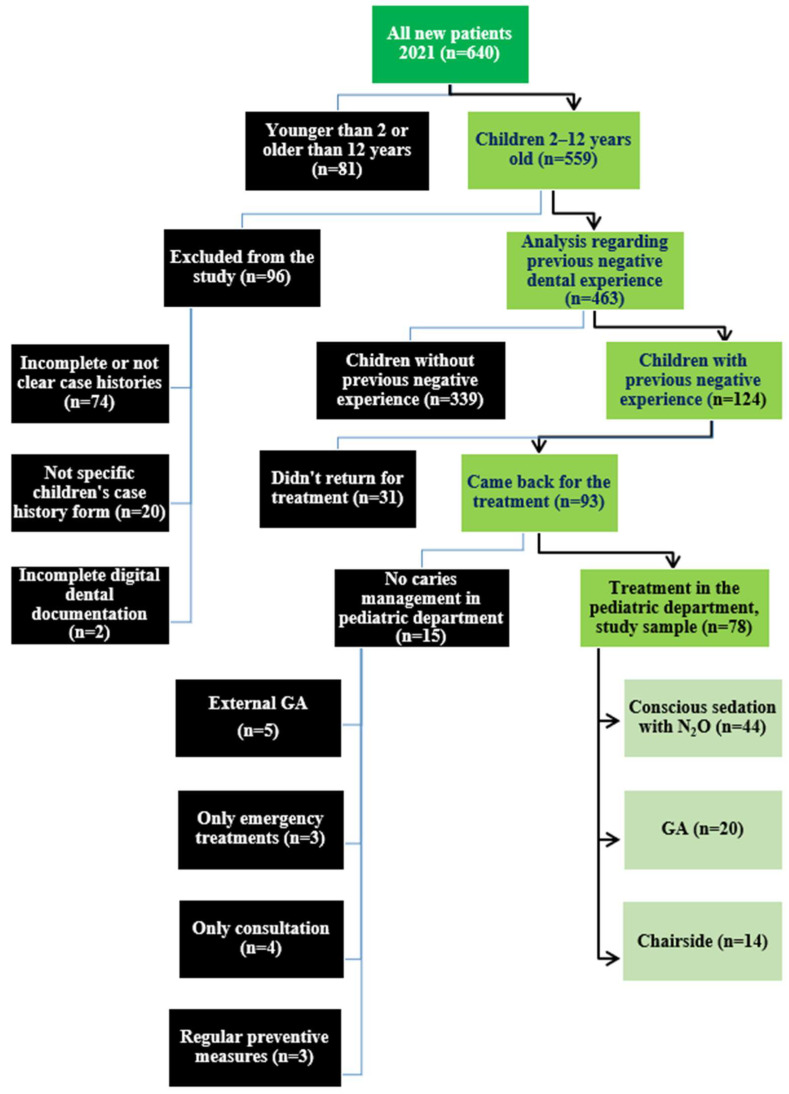
Flow chart of the study sample from initial screening to final included sample. Green boxes represent inclusion criteria. Black boxes indicate exclusions, and light green boxes show the final study sample treated in different treatment settings (nitrous oxide sedation, general anesthesia or chairside).

A total of 640 new patients were registered at the Department of Pediatric Dentistry between 1 January 2021 and 31 December 2021, as described in the flowchart (Figure 1).

The final study sample consisted of 78 new patients aged 2–12 years who presented to the department with a history of negative dental experiences and treatment needs, all of whom completed dental rehabilitation within 18 months. Of the 20 patients treated under GA, seven had initially attempted N_2_O but were unable to complete treatment, necessitating conversion to the GA pathway.

### 3.1. Characteristics of the Study Sample

The anamnestic, clinical, and behavioral characteristics of the study sample are summarized in Table 1 and Table 2.

### 3.2. Performed Dental Treatment for the Study Sample

Table 3 presents the dental treatments performed across the three patient groups during the 18-month follow-up. The mean duration to complete treatment was shortest in the N_2_O group (129 ± 119.9 days), followed by the GA group (152 ± 147.4 days), and longest in the chairside group (237 ± 162.8 days).

Stainless steel crowns were placed in 95% of GA, 52% of N_2_O, and 50% of chairside patients. Pulp therapy was more frequent under GA (35%), compared to 9% in N_2_O and 7% in chairside patients. Restorative fillings were most common in the chairside group (57%), followed by GA (50%) and N_2_O (39%).

Following extractions, 30% of N_2_O patients received removable space maintainers (SMs) and 18% received fixed SMs. In the GA group, 10% received removable and 65% received fixed SMs, while only one chairside patient received a fixed SM.

**Figure 2:**
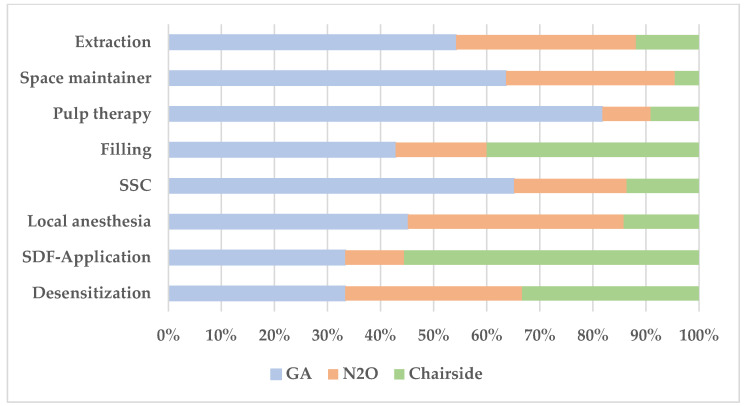
Distribution of types of dental treatments performed across different treatment settings.

Figure 2 presents the mean number of each type of dental treatment performed per child across treatment settings as well as the relative distribution to the different treatment modalities.

### 3.3. Predictors for Dental Treatment Under General Anesthesia

The second aim of the study was to identify specific predictive factors for treatment under GA. Comparative analyses were performed across children managed under GA, N_2_O, and chairside treatment. In univariate analysis, age, dmft, dt, cooperation level, and dmft > age (years) differed significantly in children treated under GA (all *p* < 0.05, Table 4).

No significant differences were observed for mean DMFT or DT scores between groups. Gender distribution, systemic health status, chief complaint, emergency visits, referral patterns, pain history, pain at first visit and parental willingness to pay for extra desensitization visits or to leave during treatment showed also no statistically significant differences across the groups (all *p* > 0.05, Table 1 and Table 2).

To identify predictors for the need for dental treatment under GA for this selected patient’s sample, all variables that showed significant differences (Table 4), were included in the logistic regression analysis. However, in the multivariate logistic regression model, none of these variables remained statistically significant after mutual adjustment (*p* > 0.05, Table 5).

## 4. Discussion

One fourth of all new patients aged 2–12 years presenting to the university dental clinic in 2021 had a previous negative dental experience that led to treatment interruption, resulting either in referral by the primary dentist or in the parents’ decision to change dentists. Previous negative dental experiences are well-recognized predictors of dental anxiety and treatment avoidance [10,11,12,13]. Such experiences may contribute to the high proportion of untreated primary teeth observed in this cohort. The mean dmft was 4.5, reflecting a high caries risk compared to national data for this age group in Germany. Notably, 76% of the primary teeth in our sample remained untreated before visiting our department, far exceeding the 43% untreated rate reported in the national survey for the same age group [1].

Among the study sample, the most common chief complaint was caries in the primary dentition, and the main reason for referral was difficulty with behavior management, often accompanied by a request for treatment completion using pharmacological interventions. Despite this background, dental caries management was possible without general anesthesia (GA) in 74% of cases. Nitrous oxide (N_2_O) was used more frequently for invasive procedures such as extractions and administration of local anesthesia, whereas non-restorative cavity control (NRCC) with silver diamine fluoride (SDF), fillings using minimally invasive techniques, and selective caries removal were primarily applied chairside. GA was performed in approximately 26% of the children, particularly among those of younger age, with higher dmft scores, and with lower cooperation levels, when complete rehabilitation with more complex treatments such as SSC placement and pulp therapy was indicated.

Younger age, higher dt/dmft values, lower cooperation scores on the Frankl scale, and a dmft score exceeding the child’s chronological age were associated with a greater likelihood of requiring treatment under GA. However, in the multivariable regression analysis, none of the examined factors emerged as significant independent predictors for the need for GA in this study population.

These findings are consistent with other studies showing that dental anxiety and behavior management problems in children with restorative treatment needs are the main reasons for referral to pediatric dentists and for requests for sedation [24,25]. The observed association between younger age and higher dmft scores with the need for GA mirrors prior studies, which have consistently reported that young age and extensive treatment needs increase the likelihood of requiring sedation or anesthesia [26,27]. Similarly, the higher proportion of uncooperative children in the GA group aligns with previous work identifying lack of cooperation as the most common reason for dental treatment under GA [28].

Other studies have proposed that a dmft score exceeding the child’s chronological age (dmft > age) may serve as an objective predictor of the need for dental treatment under GA [29,30,31]. In our study, this factor showed an association in unadjusted analyses but did not remain significant in the multivariable model.

The lack of identifiable predictors for GA emphasizes that, with individualized and well-considered treatment planning, even young children with traumatic dental experiences can frequently be treated without general anesthesia.

These findings suggest that some factors like age and increased caries experience may be associated together and confirm the complexity of decision-making in pediatric dentistry with the necessity of individual case specific assessment. Several studies emphasize the individual decision-making for selecting the treatment modalities in children based on cooperation level and treatment need. In children with mild to moderate levels of dental anxiety and without emergency treatment needs, simple behavior management techniques and establishing a sense of trust and control during dental treatment may be useful. In the presence of acute treatment need, dental pain and deterioration of oral health, behavior management techniques should be supported with pharmacological interventions. In the presence of sever dental anxiety [32] in addition to emergency treatment needs, dental intervention under general anesthesia is indicated [33].

Opposite to our findings, low cooperation has been reported as a significant predictor for treatment under GA in other studies [31]. The absence of independent predictive power for this variable in our regression model may reflect the multifactorial nature of cooperation in pediatric dentistry. Behavioral responses in children are shaped by a complex interplay of dental fear, previous experiences, cognitive and emotional development, family dynamics, and environmental influences, many of which are difficult to isolate or fully capture in retrospective analyses [34,35].

The different approaches to caries management applied across treatment settings in our study align with previous findings and emphasize the importance of individualized treatment planning in pediatric patients. Several studies indicate that in very young children with early childhood caries (ECC) without pulpal involvement, conservative strategies—such as oral hygiene improvement, topical fluoridation, non-restorative cavity control (NRCC), caries inactivation with SDF [36], or minimally invasive approaches including atraumatic restorative treatment (ART) and non- or selective caries removal [37]—can preserve pulp vitality, extend tooth longevity, and prevent the need for more complex pulpal treatments. In high–caries-risk children, the placement of stainless steel crowns (SSC) using the Hall Technique represents a cost-effective and easily applicable option for caries management [36,37,38,39,40], thereby reducing the likelihood of requiring GA. Importantly, these approaches can be performed without local anesthesia and without the use of a dental drill, both of which are known triggers for dental anxiety in children [13,41,42]. However, when ECC is accompanied by pulpal symptoms in very young children, complete dental rehabilitation under GA remains the recommended approach [38].

There is limited global or national information regarding the prevalence of complete dental rehabilitation of children under GA, and no national data are available for Germany. Only two studies have reported population-level estimates: one from Dubai, reporting a GA prevalence of 6.1% with a mean age of 4.9 years [43], and one from Belgium, reporting a prevalence of 1.59% with a mean age of 5.29 years [44]. Both studies, however, were conducted in specialized pediatric hospital settings and therefore may not be directly comparable to our university outpatient clinic population. The considerably higher prevalence of GA in our sample (26%) may be partly explained by the high proportion of children with previous negative dental experiences, which is likely to increase dental anxiety, reduce cooperation, and consequently elevate the need for pharmacological management. Given the lack of standardized prevalence data and the potential influence of negative past experiences, further well-designed studies—ideally randomized controlled trials—are needed to determine whether negative dental experiences causally increase the likelihood of requiring GA in pediatric patients.

The findings of our study emphasize the importance of early behavioral assessment and individualized treatment planning in pediatric dental patients, particularly those with high dmft/dft values or a history of dental fear and anxiety (DFA). When dental visits are initiated early, before the development of advanced lesions or severe anxiety, children can be more effectively managed both behaviorally and clinically.

Clinicians are encouraged to exhaust non-pharmacological and behavioral interventions, as well as minimally invasive caries management strategies, before proceeding to general anesthesia (GA). The high parental willingness observed in our study (85%) to invest in desensitization sessions indicates that families are generally receptive to such non-pharmacological approaches, which should be integrated systematically into treatment planning.

Referral to GA should be made more selectively, taking into account behavioral, parental, and contextual factors that may predict successful chairside treatment. When a child presents with severe anxiety, behavioral challenges, or complex treatment needs beyond the capabilities of a general practitioner, timely referral to a pediatric dental specialist is recommended. Specialists are better equipped to assess whether alternative treatment approaches can be employed or whether pharmacological interventions, including GA, are truly necessary. Overall, appropriate early assessment, individualized care, and judicious use of specialist services may help reduce unnecessary GA while ensuring safe and effective treatment for children with negative dental experiences.

In the university clinic, GA is reserved for cases where it is truly necessary. We acknowledge that children who did not return were possibly from those whose parents who preferred to complete treatment under GA in a single session at a private practice [43]. This could lead to an overestimation of GA use in such settings. On the other hand, there are very few dentists in our region who provide GA options for children, so we assume this number is low.

This study provides a thorough and comprehensive overview of the management of dental problems in a particularly vulnerable and challenging pediatric population—children requiring further treatment after having experienced previous negative dental experiences. The retrospective design offers the advantage of assessing clinical pathways without influencing treatment decisions, thereby reflecting real-life clinical conditions. The inclusion of multivariable analysis further strengthens the conclusions regarding potential predictors for GA and underscores the multifactorial nature of treatment decision-making in pediatric dentistry. Moreover, the detailed reporting of treatment modalities used under GA, with conscious sedation and chairside provides valuable practical insights for clinicians managing children with negative dental experiences, supporting the development of individualized and minimally invasive treatment strategies.

Although the study was performed in Germany, the focus on high-risk children suggests that these findings may be highly likely generalizable to other populations, including those in societies with comparable or even less favorable socioeconomic conditions, where children may be at similar or higher risk of severe caries. The findings of this study may also be generalizable to clinical settings where pediatric dentists are not readily available or where access to general anesthesia is limited due to lack of equipment or specialized facilities. In such contexts, the results can support general practitioners in selecting alternative, evidence-based approaches for managing caries in young or uncooperative children.

Several methodological limitations of this study should be acknowledged. First, the retrospective design limits the ability to draw causal inferences. Second, the relatively small size of the GA group may have reduced statistical power, particularly in multivariable analyses comparing subgroups. Prospective studies with larger cohorts and greater statistical power are recommended to validate and strengthen the evidence presented here. Finally, the exclusion of children who did not return for follow-up may introduce selection bias, as these patients could represent a group with more severe behavioral challenges. Future studies should investigate whether and where these children continued their dental treatment to better understand long-term outcomes and identify potential gaps in care.

**Clinical implications**

- The results encourage clinicians to exhaust non-pharmacological and behavioral interventions as well as minimally invasive caries management before proceeding to GA.
- Referral to GA should be made more selectively by identifying behavioral, parental, or contextual factors that predict successful chairside treatment.
- Specialist care may be able to reduce unnecessary GA use in pediatric dentistry.

## 5. Conclusions

Minimally invasive caries management, combined with appropriate behavior guidance and conscious sedation when indicated, can drastically reduce the need for general anesthesia in children, even among those with dental anxiety or previous negative dental experience.

## Figures and Tables

**Table 1:** Anamnestic characteristics of the study sample differentiated by treatment setting.

Treatment Setting n (%)		Total 78 (100%)	N_2_O 44 (56.4%)	GA 20 (25.6%)	Chairside 14 (18%)	* p * Value *
Age (y) (mean ± SD)		6.7 ± 2.2	7.3 ± 1.7	5.13 ± 1.9	6.9 ± 2.8	0.001 **
Gender n (%)	Female	37 (47.4%)	20 (45%)	11 (55%)	6 (42.9%)	0.724
	Male	41 (52.6%)	24 (55%)	9 (45%)	8 (57.1%)	
Known diseases n (%)	No	69 (88.5%)	39 (88.6%)	16 (80%)	14 (100%)	0.199
	Yes	9 (11.5%)	5 (11.4%)	4 (20%)	0	
Chief complaint n (%)	Caries	69 (88.5%)	40 (90.9%)	18 (90%)	11 (78.6%)	0.097
	Trauma	2 (2.6%)	1 (2.3%)	0	1 (7.1%)	
	Orthodontic	1 (1.3%)	1 (2.3%)	0	0	
	Checkup	2 (2.6%)	0	0	2 (14.3%)	
	Others	4 (5.1%)	2 (4.5%)	2 (10%)	0	
Reason of referral n (%)	No	30 (38.5%)	18 (40.9%)	5 (25%)	7 (50%)	0.795
	Lack of cooperation	28 (35.9%)	14 (31.8%)	10 (50%)	4 (28.6%)	
	N_2_O	10 (12.8%)	7 (15.9%)	2 (10%)	1 (7.1%)	
	GA	7 (8.9%)	4 (9.1%)	2 (10%)	1 (7.1%)	
	Others	3 (3.8%)	1 (2.3%)	1 (5%)	1 (7.1%)	
Pain history n (%)	No	25 (32.1%)	16 (36.4%)	5 (25%)	4 (28.6%)	0.634
	Yes	53 (67.9%)	28 (63.6%)	15 (75%)	10 (71.4%)	
Parents ready to leave the treatment room n (%)	No	16 (20.5%)	8 (18.2%)	7 (35%)	1 (7.1%)	0.304
	Yes	55 (70.5%)	31 (70.4%)	12 (60%)	12 (85.8%)	
	No Information	7 (9%)	5 (11.4%)	1 (5%)	1 (7.1%)	
Parents ready to come and pay for extra desensitization visits n (%)	No	2 (2.6%)	2 (4.6%)	0	0	0.246
	Yes	66 (84.6%)	39 (88.6%)	15 (75%)	12 (85.7%)	
	No Information	10 (12.8%)	3 (6.8%)	5 (25%)	2 (14.3%)	

* *p*-values calculated by comparing distribution between patients treated under N_2_O sedation, GA and chairside. ** *p*-value ≤ 0.05 considered statistically significant. One-way ANOVA or Kruskal–Wallis test was applied as appropriate.

**Table 2:** Dental and behavioral characteristics of the study sample differentiated by treatment setting.

Treatment Setting n (%)		Total 78 (100%)	N_2_O 44 (56.4%)	GA 20 (25.6%)	Chairside 14 (18%)	* p * Value *
Emergency visit n (%)	Yes	4 (5.1%)	3 (6.8%)	1 (5%)	0	0.602
	No	74 (94.9%)	41 (93.2%)	19 (95%)	14 (100%)	
Reason of referral n (%)	No	30 (38.5%)	18 (40.9%)	5 (25%)	7 (50%)	0.795
	Lack of cooperation	28 (35.9%)	14 (31.8%)	10 (50%)	4 (28.6%)	
	N_2_O	10 (12.8%)	7 (15.9%)	2 (10%)	1 (7.1%)	
	GA	7 (9%)	4 (9.1%)	2 (10%)	1 (7.1%)	
	Others	3 (3.8%)	1 (2.3%)	1 (5%)	1 (7.1%)	
Cooperation in first visit: Frankl scale n (%)	1	16 (20.5%)	5 (11.4%)	10 (50%)	1 (7.1%)	0.016 **
	2	7 (8.9%)	3 (6.8%)	2 (10%)	2 (14.3%)	
	3	36 (46.2%)	24 (54.6%)	6 (30%)	6 (42.9%)	
	4	16 (20.5%)	10 (22.7%)	1 (5%)	5 (35.7%)	
	No Information	3 (3.9%)	2 (4.5%)	1 (5%)	0	
Baseline dt (mean ± SD)		3.4 ± 2.7	3.3 ± 2.4	5.6 ± 2.6	1.9 ± 2.4	0.000 **
Baseline dmft (mean ± SD)		4.5 ± 3	4.2 ± 2.8	6.2 ± 2.7	2.8 ± 2.8	0.002 **
Baseline DT (mean ± SD)		0.4 ± 1	0.4 ± 1.1	0.6 ± 1.1	0.4 ± 1	0.932
Baseline DMFT (mean ± SD)		0.6 ± 1.2	0.6 ± 1.3	0.6 ± 1.1	0.6 ± 1	0.942
Pain at first visit, n (%)	No	49 (62.8%)	29 (65.9%)	10 (50%)	10 (71.4%)	0.362
	Yes	29 (37.2%)	15 (34.1%)	10 (50%)	4 (28.6%)	

* *p*-values calculated by comparing distribution between patients treated under N_2_O sedation, GA and chairside. ** *p*-value ≤ 0.05 considered statistically significant. One-way ANOVA or Kruskal–Wallis test was applied as appropriate.

**Table 3:** Dental treatment profile of the study sample differentiated by treatment pathway.

Treatment Profile		N_2_O (n = 44)	GA (n = 20)	Chairside (n = 14)
Number of desensitization visits n (%)	0	22 (50%)	12 (60%)	9 (64.3%)
	1	20 (45.5%)	6 (30%)	3 (21.4%)
	2	2 (4.5%)	2 (10%)	2 (14.3%)
Number of N_2_O-sessions n (%)	0	-	13 (65%)	14 (0%)
	1	20 (45.5%)	5 (25%)	-
	2	15 (34.1%)	2 (10%)	-
	3	6 (13.6%)	-	-
	4	2 (4.5%)	-	-
	5	1 (2.3%)	-	-
Number of SDF applications n (%)	0	38 (86.4%)	16 (80%)	8 (57.1%)
	1	6 (13.6%)	3 (15%)	5 (35.7%)
	2	-	-	1 (7.1%)
	3	-	1 (5%)	-
Number of teeth extracted n (%)	0	5 (11.4%)	-	11 (78.6%)
	1	16 (36.4%)	4 (20%)	2 (14.3%)
	2	9 (20.4%)	4 (20%)	-
	3	8 (18.1%)	2 (10%)	-
	4 or more	6 (13.6%)	10 (50%)	1 (7.1%)
Number of injected local anesthesia n (%)	0	4 (9.1%)	-	10 (71.4%)
	1	13 (29.5%)	3 (15%)	3 (21.4%)
	2	11 (25%)	8 (40%)	-
	3	8 (18.2%)	4 (20%)	-
	4 or more	8 (18.2%)	5 (25%)	1 (7.1%)
Number of SSC n (%)	0	21 (47.7%)	1 (5%)	7 (50%)
	1	5 (11.4%)	2 (10%)	2 (14.3%)
	2	8 (18.2%)	2 (10%)	5 (35.7%)
	3	2 (4.5%)	1 (5%)	-
	4 or more	8 (18.2%)	14 (70%)	-
Number of pulp therapies n (%)	0	40 (90.9%)	13 (65%)	13 (92.9%)
	1	3 (6.8%)	1 (5%)	1 (7.1%)
	2	1 (2.3%)	3 (15%)	-
	3	-	1 (5%)	-
	4	-	2 (10%)	-
Number of fillings n (%)	0	27 (61.4%)	10 (50%)	6 (42.9%)
	1	10 (22.7%)	2 (10%)	3 (21.4%)
	2	6 (13.6%)	3 (15%)	2 (14.3%)
	3	1 (2.3%)	2 (10%)	1 (7.1%)
	4 or more	-	3 (15%)	2 (14.3%)
Type of space maintainer (%)	0	23 (52.3%)	5 (25%)	13 (92.9%)
	Removable	13 (29.5%)	2 (10%)	-
	fixed	8 (18.2%)	13 (65%)	1 (7.1%)
Average treatment time (days)	-	129 ± 120	152 ± 147	237 ± 163

**Table 4:** Results of the logistic regression analysis (univariate score tests) on factors affecting the different treatment pathways.

Factor		N_2_O (n = 44)	GA (n = 20)	Chairside (n = 14)	* p * Value *
Age (y)	(mean ± SD)	7.3 ± 1.7	5.1 ± 1.9	6.9 ± 2.8	<0.001 **
dmft	(mean ± SD)	4.2 ± 2.8	6.2 ± 2.7	2.8 ± 2.8	0.034 **
dt	(mean ± SD)	3.3 ± 2.4	5.6 ± 2.6	1.9 ± 2.4	0.005 ***
Frankl scale n (%)	1	5 (11.4%)	10 (50%)	1 (7.1%)	0.016 ****
	2	3 (6.8%)	2 (10%)	2 (14.3%)	
	3	24 (54.6%)	6 (30%)	6 (42.9%)	
	4	10 (22.7%)	1 (5%)	5 (35.7%)	
	No information	2 (4.5%)	1 (5%)	-	
Children with dmft > age n (%)		12 (27.3%)	14 (70%)	2 (14.3%)	0.007 ****

*: *p*-value ≤ 0.05 considered statistically significant. **: One-way ANOVA. ***: Kruskal–Wallis test. ****: Pearson Chi-square test.

**Table 5:** Odds ratio for having GA for dental treatments in the study population using logistic regression analysis (multivariate score tests).

Logistic Regression		*p* Value *	OR **	CI (95%) ***
Age (y)		0.18	0.688	0.398–1.188
dmft		0.531	0.815	0.430–1.545
dt		0.415	1.318	0.679–2.557
Frankl scale	1	0.244	Ref.	
	2	0.421	0.348	0.027–4.538
	3	0.093	0.238	0.044–1.273
	4	0.080	0.102	0.008–1.308
Children with dmft > age n (%)		0.582	2.056	0.158–26.797

*: *p*-value ≤ 0.05 considered statistically significant. **: odds ratio. ***: Confidence intervals.

## Data Availability

The data underlying this study are not publicly available due to privacy and data protection regulations under §§37–37d of the *Landeskrankenhausgesetz Mecklenburg-Vorpommern (LKHG M-V)*. Data may be made available from the corresponding author upon reasonable request and with permission from the institutional ethics committee of the University Medicine Greifswald and the local data protection officer.

## Abbreviations

The following abbreviations are used in this manuscript:

ART	Atraumatic restorative treatment
BMT	Behavior management techniques
DFA	Dental fear and anxiety
DMFT	Decayed, missing, filled teeth (permanent dentition)
DT	Decayed teeth (permanent dentition)
dmft	Decayed, missing, filled teeth (primary dentition)
dt	Decayed teeth (primary dentition)
ECC	Early childhood caries
GA	General anesthesia
LA	Local anesthesia
N_2_O	Nitrous oxide (inhalation sedation)
NRCC	Non-restorative caries control
OHRQoL	Oral health-related quality of life
SaC index	Specific affected caries index
SiC index	Significant caries index
SDF	Silver diamine fluoride
SM	Space maintainer
SSC	Stainless steel crown
WHO	World Health Organization
